# Anaesthetic Management of CHARGE Syndrome with Anorectal Malformation in an Infant: A Case Report

**DOI:** 10.4274/TJAR.2026.262595

**Published:** 2026-08-28

**Authors:** Nidhi Singh, Geeta Kamal, Charu Charu

**Affiliations:** 1 Chacha Nehru Bal Chikitasalaya Raja Ram Kohli Marg Department of Paediatric Anaesthesia, Delhi, India

**Keywords:** Airway management, anorectal malformation, CHARGE syndrome, paediatric anaesthesia, perioperative care, videolaryngoscopy

## Abstract

CHARGE syndrome is a rare congenital disorder with multisystem involvement, posing significant anaesthetic challenges. We report the perioperative management of an 11-month-old male who was diagnosed with anorectal malformation and CHARGE syndrome and who underwent posterior sagittal anorectoplasty. Key anaesthetic considerations included craniofacial asymmetry, facial nerve palsy, and anticipated airway difficulties. Inhalational induction with careful preoxygenation, video laryngoscopy, and caudal analgesia facilitated safe anaesthesia. Post-operative airway compromise was managed with continuous positive airway pressure, and the patient recovered without further complications or intervention. Ultrasound guidance may facilitate identification of relevant anatomy and real-time visualization of injectate spread during caudal block. This case highlights the importance of meticulous pre-anaesthetic planning and multidisciplinary evaluation in children with CHARGE syndrome.

**Main Points**

• CHARGE syndrome presents unique anaesthetic challenges, especially airway management.

• Preoxygenation and confirmation of mask ventilation are crucial before intubation.

• Video laryngoscopy facilitates safe intubation in patients with craniofacial anomalies.

• Post-operative airway compromise can occur even after a smooth intra-operative course.

• Multidisciplinary evaluation (ear, nose and throat, ophthalmology, and cardiology) is essential.

## Introduction

Anorectal malformation (ARM) refers to a range of conditions characterised primarily by an absent or abnormal anal opening. The incidence is 1 in 5000 live births and is more common in males.^1^ The term “CHARGE” is an acronym for a group of clinical features, including Coloboma, heart defects, choanal atresia, retardation (of growth and/or development), Genitourinary malformations, and Ear abnormalities. The majority of patients have a single mutation in the *CHD7* gene.^2^ CHARGE syndrome occurs in 1 in 10,000 live births and affects males and females equally.^3^ From an anaesthetic perspective, these patients pose considerable perioperative challenges, including potential airway difficulty, congenital cardiac lesions, and neurological abnormalities.

We report the anaesthetic management of an 11-month-old infant with ARM and CHARGE syndrome, who was posted for anorectoplasty, emphasising its perioperative concerns.

## Case Report

An 11-month-old male infant weighing 5.1 kg and diagnosed with ARM and CHARGE syndrome was scheduled for posterior sagittal anorectoplasty.

**Figure 1:**
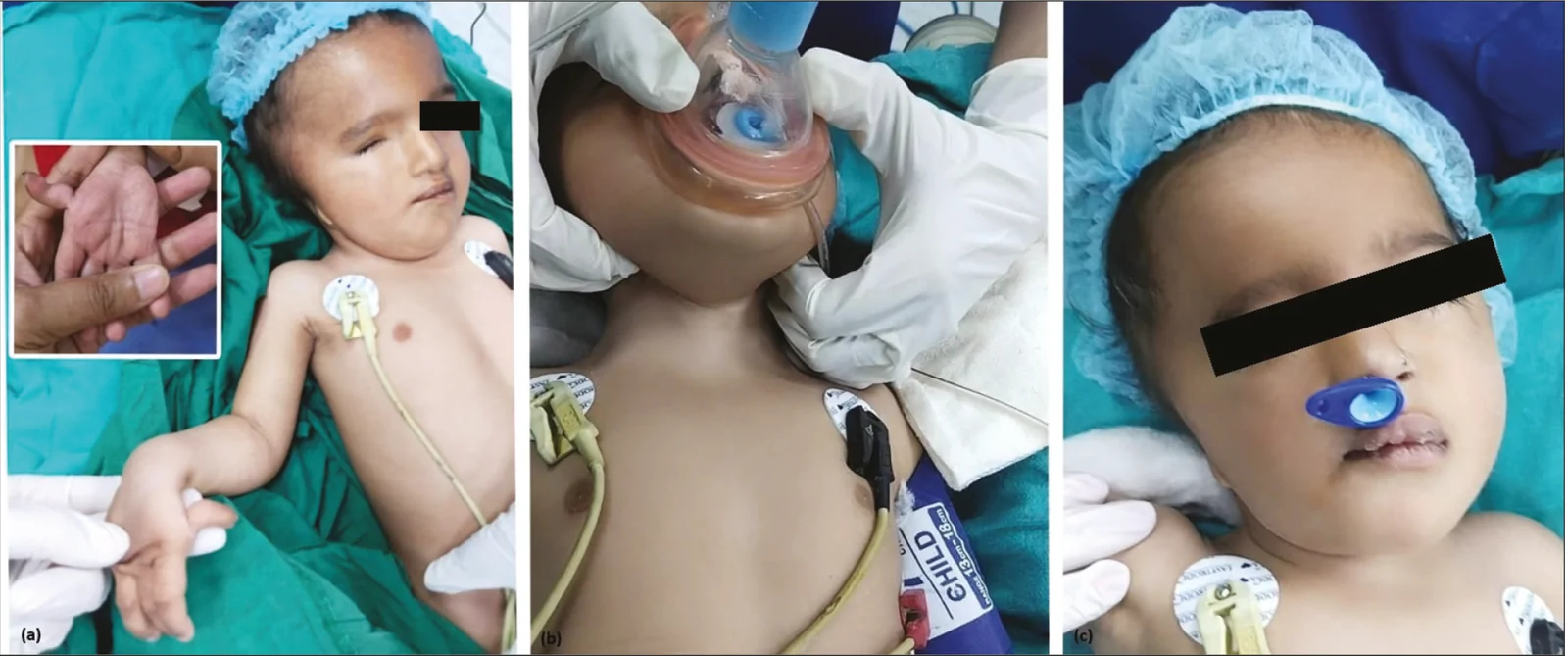
Clinical features of CHARGE syndrome. (a) Retrognathia, right-sided facial palsy, right anotia, right microphthalmos, right hanging thumb polydactyly, J-shaped palmar crease (sub-image). (b) Oral airway and jaw-thrust to ensure adequate mask ventilation. (c) The appropriately sized nasopharyngeal airway maintained a patent airway in the early post-operative period.

The patient was born at term with a birth weight of 2.5 kg and was diagnosed with an imperforate anus. On day 1 of life, he underwent a sigmoid colostomy at another hospital. There was no significant antenatal or postnatal history. Currently, his developmental milestones are delayed. His weight was below the 3^rd^ percentile, suggesting failure to thrive. The child exhibited multiple congenital anomalies consistent with CHARGE syndrome, including left iris coloboma, right facial nerve palsy, right anotia, right microphthalmos, right hanging thumb polydactyly, J-shaped palmar crease, and growth retardation (Figure 1). The presence of retrognathia, a short neck, and facial abnormalities led us to anticipate a difficult airway, particularly with regard to mask ventilation.

The examination revealed a heart rate of 120 beats/min, a respiratory rate of 20 breaths/min, and a blood pressure of 90/50 mmHg. Oxygen saturation was 98% on room air. Respiratory and cardiovascular examinations were normal. Routine laboratory investigations were within normal limits. Two-dimensional echocardiography and radiographs of the chest and lumbosacral spine revealed no abnormalities. Ophthalmology and ear, nose and throat evaluations were performed, and imaging of the brain and temporal bone was recommended for follow-up. Written informed consent for the planned anaesthesia and surgery, as well as for publication of the case details, was obtained from the patient’s parents.

### Anaesthetic Management

After the patient’s fasting status was confirmed, the patient was taken to the operating theatre, and standard monitors were applied.

**Figure 2:**
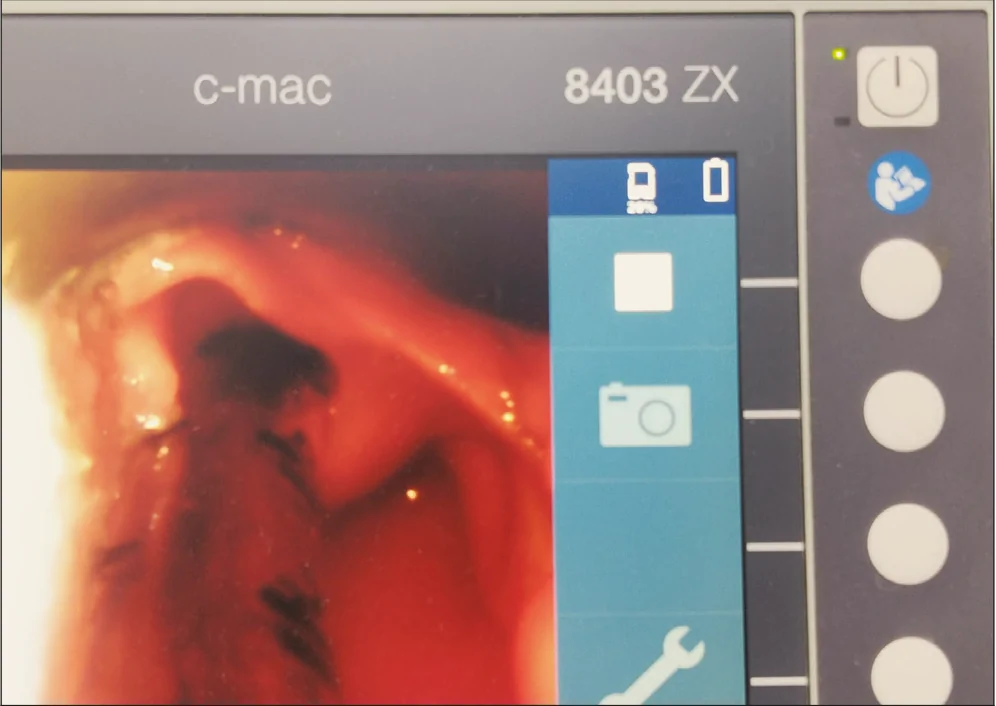
POGO score of 50% seen on C-MAC video laryngoscope with oral ETT secured. POGO, percentage of glottic opening; ETT, endotracheal tube.

**Figure 3:**
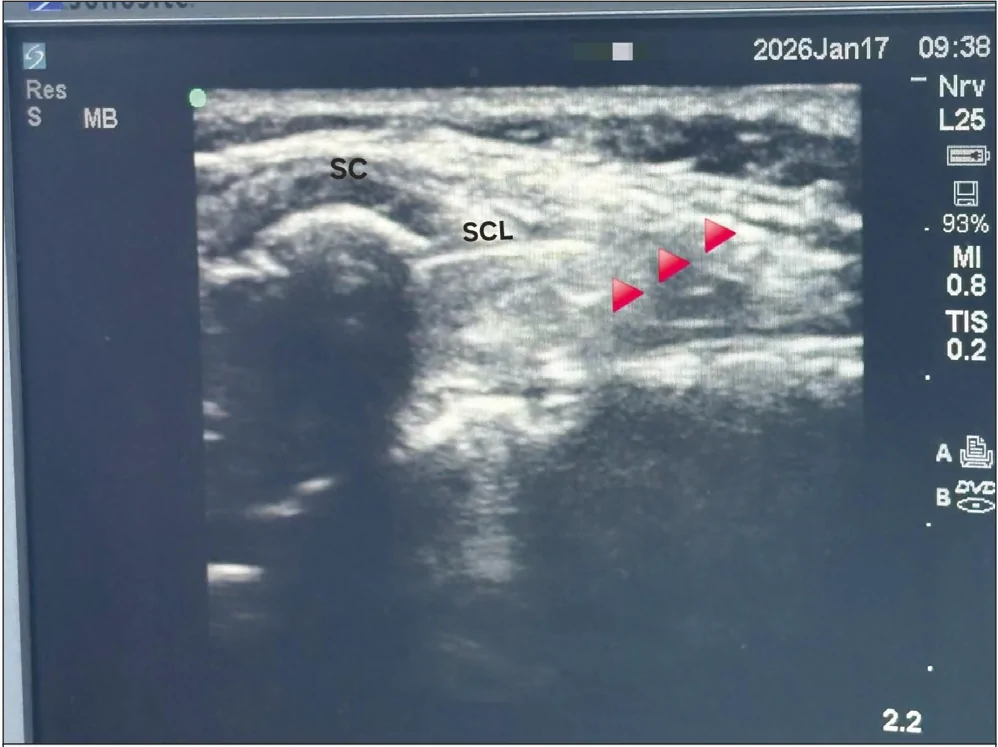
Ultrasonographic image of the caudal space depicting sacral cornu (SC) and sacrococcygeal ligament (SCL) with a needle in the caudal space (marked with red arrows).

The difficult airway cart was kept ready for use. Inhalational induction of anaesthesia was performed using sevoflurane in 100% oxygen. A 22-G intravenous (IV) cannula was secured, and IV glycopyrrolate (5 μg kg^-1^) and fentanyl (1 μg kg^-1^) were given. Adequate mask ventilation was established with an oral airway. Nasal patency was confirmed by the gentle passage of a catheter. A smooth video laryngoscopy using a size 2 Macintosh blade on the C-MAC revealed a percentage of glottic opening of 50% (Figure 2). The video laryngoscopy showed normal supraglottic and glottic anatomy and no airway collapse. As tracheomalacia is an associated airway anomaly in CHARGE syndrome, dynamic bronchoscopy is the gold standard for diagnosis. In the absence of preoperative stridor, recurrent desaturation, persistent wheezing, or any history suggestive of significant airway compromise, we avoided further invasive airway evaluation. After administering IV atracurium (0.5 mg kg^-1^) and ventilating for 3 minutes, an oral uncuffed endotracheal tube (ETT) of 4.5 mm internal diameter was secured. The Correct placement of the ETT was confirmed by bilateral auscultation and capnography. Anaesthesia was maintained with pressure-controlled ventilation using a 50:50 oxygen and air mixture at a minimal alveolar concentration of 1-1.5. For regional analgesia, an ultrasound-guided caudal epidural block was administered in the left lateral position, using 5 mL of isobaric bupivacaine (0.25%) (Figure 3).

The patient was positioned in the prone jackknife position, with careful padding of the eyes and pressure points. Intraoperatively, a recto-bulbar (urethral) fistula was identified, with the preoperatively secured urinary catheter malpositioned within the fistulous tract. Later, a suprapubic urinary catheter was secured. The surgical time was 90 minutes. Intra-operative vital signs remained stable.

At the end of surgery, IV paracetamol 15 mg kg^-1^ was given, and neuromuscular blockade was reversed with neostigmine (50 μg kg^-1^) and glycopyrrolate (10 μg kg^-1^). After extubation, the patient developed suprasternal retraction and accessory muscle use. This was managed using a nasopharyngeal airway, continuous positive airway pressure at 5-10 cm H_2_O, and 100% oxygen. The patient stabilised within minutes and was transferred to the recovery room. No further complications occurred.

## Discussion

ARM result in an abnormal anal opening with a fistulous tract between the rectum and the urinary system (recto-urethral fistula in approximately 70% of cases) in males, or a fistulous connection with the reproductive system in females.^1^

The cause of ARM is unknown, but genetic factors play a significant role. Almost 60% of cases have associated vertebral, anorectal, cardiac, tracheoesophageal fistula/oesophageal atresia, renal, and limb (VACTERL) defects. ARM has been reported in association with trisomy 8 mosaicism, Down syndrome, and Fragile X syndrome.^4^ The association between CHARGE syndrome and ARM is less commonly reported. Dworschak et al.^5^ have reported CHARGE syndrome, resulting from de novo variants, as a rare monogenic cause of syndromic ARM.

The diagnosis of CHARGE syndrome is primarily clinical, as first described by Blake et al.^6^ and later modified by Verloes^7^. According to Verloes’^7^ criteria, our patient met one major criterion (coloboma) and two minor criteria (facial nerve palsy and right ear anotia). Notable phenotypic features include developmental delay, polydactyly, and a J-shaped palmar crease.The presence of recto-bulbar fistula is consistent among male patients with syndromic ARM.

The current guidelines for difficult airways in infants recommend video laryngoscopy as the first-line intubation technique. This approach reduces the number of intubation attempts and the consequent complications.^8^

Post-extubation respiratory distress may have been due to upper airway oedema caused by prone positioning. A similar event was reported by Kumar and Venkatesh^9^ who suspected that the cause was glossoptosis and pharyngeal collapse due to underlying hypotonia or cranial nerve dysfunction.

The application of difficult-airway guidelines and regional analgesia provided safe anaesthesia for this syndromic infant. ARM is typically associated with VACTERL anomalies, but our case emphasises the anaesthetic challenges and considerations arising from its rare association with CHARGE syndrome.

## Conclusion

This case report provides valuable insights into thorough preoperative evaluation, multidisciplinary management, and structured perioperative strategies for a syndromic infant, particularly regarding difficult airway management.

## Ethics

**Informed Consent:** Written informed consent for the planned anaesthesia and surgery, as well as for publication of the case details, was obtained from the patient’s parents.
